# RuCl_3_·3H_2_O Catalyzed Reactions: Facile Synthesis of Bis(indolyl)methanes under Mild Conditions

**DOI:** 10.3390/molecules16053855

**Published:** 2011-05-09

**Authors:** Hong-En Qu, Chen Xiao, Ning Wang, Kai-Hui Yu, Qiao-Sheng Hu, Liang-Xian Liu

**Affiliations:** Department of Chemistry and Biology, Gannan Normal University, Ganzhou, Jiangxi 341000, China; Email: quhongen-2003@163.com (H.-E.Q.); xiaocen1219@163.com (C.X.); wangning198415@126.com (N.W.); ykh506@163.com (K.-H.Y.)

**Keywords:** bis(indolyl)methane, RuCl_3_·3H_2_O, aldehyde, indole

## Abstract

RuCl_3_·3H_2_O was found to be an effective catalyst for reactions of indoles, 2-methylthiophene, and 2-methylfuran with aldehydes to afford the corresponding bis(indolyl)methanes, bis(thienyl)methanes, and bis(fur-2-yl)methanes in moderate to excellent yields. Experimental results indicated that mono(indolyl)methanol is not the reaction intermediate under these reaction conditions.

## 1. Introduction

Indoles and their derivatives are known to possess various pharmacological and biological properties, including antibacterial, cytotoxic, antioxidative, and insecticidal activities [1,2]. Furthermore, bis(indolyl)alkanes and their derivatives constitute an important group of bioactive metabolites of terrestrial and marine origin [3,4,5,6,7,8]. During the past decade a large number of natural products containing bis(indolyl)methanes [9] and bis(indolyl)ethanes [10] have been isolated from marine sources. Consequently, a number of synthetic methods for the preparation of bis(indolyl)alkane derivatives by reacting indoles with various aldehydes and ketones in the presence of either a Lewis acid [11] or a protic acid [12,13,14], metal salts, such as In(OTf)_3_ [15], Dy(OTf)_3_ [16,17], Ln(OTf)_3_ [18], and CeCl_3_·7H_2_O [19,20], and molecular iodine [21,22], as well as solid acidic catalysts [23,24,25], such as clays and Zeolites, have been reported in the literature. In addition, it has been reported that the reactions of indoles with various aldehydes were carried out in a protic solvent in the absence of any other catalyst to afford bis(indolyl)methanes [26]. In this study, we report a facile and efficient procedure for the synthesis of bis(indolyl)methanes, bis(thienyl)methanes, and bis(fur-2-yl)methanes under mild conditions using RuCl_3_·3H_2_O as catalyst.

## 2. Results and Discussion

In the first instance, we studied the reaction of indole with benzaldehyde as a model reaction. We found that this reaction was fast in the presence of RuCl_3_·3H_2_O (5 mol %) in ethylene glycol dimethyl ether (GDE) at room temperature, and the corresponding bis-indolylmethane was obtained in 87% yield after 30 min (Table 1, entry 6).

**Table 1:** Effect of RuCl_3_·3H_2_ Oloading *^a^*.

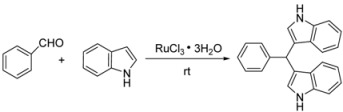			
Entry	RuCl_3_·3H_2_O [equivalents]	Solvent	Yield [%] *^b^*
1	0.1	Benzene	93
2	0.05	Benzene	92
3	0.03	Benzene	75
4	0.02	Benzene	60
5	0	Benzene	0
6	0.05	GDE *^c^*	87
7	0.05	THF	88
8	0.05	DCM	87
9	0.05	Chloroform	86
10	0.05	Acetone	83
11	0.05	Acetonitrile	89

*^a^* The reaction was performed with benzaldehyde (0.5 mmol), indole (1 mmol) and RuCl_3_·3H_2_O (0.05 mmol) in 1 mL of solvent at rt for 30 min. *^b^* Isolated yield. *^c^* Ethylene glycol dimethyl ether.

To optimize the reaction conditions, we have studied the effect of different solvents and RuCl_3_·3H_2_O loadings on the reaction of indole with benzaldehyde. The results are shown in Table 1. After examining different solvents, including THF, GDE, CH_2_Cl_2_, C_6_H_6_, acetone, acetonitrile, and CHCl_3_, benzene, with which the highest yield of 92% was obtained when using 5 mol % RuCl_3_·3H_2_O for 30 min (Table 1, entry 2), was found to be most efficient. We next examined the effect of RuCl_3_·3H_2_O loading on the reaction; good results were obtained when using 5 mol % RuCl_3_·3H_2_O (Table 1, entry 2), and there was no advantage to using more than 5 mol % RuCl_3_·3H_2_O (Table 1, entry 1), whereas the yield significantly decreased when using only 2 mol % RuCl_3_·3H_2_O (Table 1, entry 4). Without the RuCl_3_·3H_2_O catalyst, the reaction cannot be carried out. Thus, the optimum reaction conditions for the reaction were found to be 0.05 equivalents of RuCl_3_·3H_2_O, with benzene as the solvent at r.t. To explore the scope of the reaction, next various indoles were reacted with different substituted aromatic aldehydes, and the results are summarized in Table 2.

**Table 2:** RuCl_3_·3H_2_O-catalyzed reaction of indoles with aldehydes *^a^*.

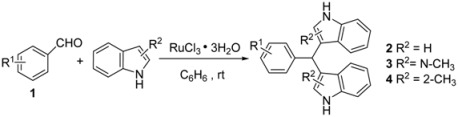					
Entry	Compounds	R_1_	R_2_	Time/h	Yield [%] *^b^*
1	**2a**	H	H	0.5	92
2	**2b**	*m*-CH_3_	H	1	77
3	**2c**	*p*-CH_3_	H	1	83
4	**2d**	*m*-OCH_3_	H	1	81
5	**2e**	*m*-Cl	H	0.5	93
6	**2f**	*o*-Br	H	0.5	89
7	**2g**	*m*-NO_2_	H	0.5	98
8	**3a**	H	*N*-CH_3_	1	75
9	**3b**	*m*-CH_3_	*N*-CH_3_	1	70
10	**3c**	*p*-CH_3_	*N*-CH_3_	1	70
11	**3d**	*m*-OCH_3_	*N*-CH_3_	1	73
12	**3e**	*m*-Cl	*N*-CH_3_	1	81
13	**3f**	*o*-Br	*N*-CH_3_	1	78
14	**3g**	*m*-NO_2_	*N*-CH_3_	1	85
15	**4a**	*m*-CH_3_	2-CH_3_	0.5	80
16	**4b**	*m*-OCH_3_	2-CH_3_	0.5	78

*^a^* The reaction was performed with aldehyde (0.5 mmol), indole (1 mmol) and RuCl_3_·3H_2_O (0.05 mmol) in 1 mL of benzene at rt. *^b^* Isolated yield.

In general, all reactions were very clean and the bis-indolylmethanes were obtained in high yields under the optimized conditions. The results have shown that substitution plays a major role in governing the reactivity of the substrate. With electron-donating substituents in the aryl aldehyde, decreased yields of products were observed (Table 2, entries 2–4, entries 9–11). For example, the reaction of *m*-methylbenzaldehyde with indole gave the corresponding product in 77% yield (Table 2, entry 2). However, the effect was reversed when electron-withdrawing groups were present in the aryl aldehyde, thus such electron-withdrawing groups (e.g., NO_2_) in the aryl aldehyde favored the reaction with indoles, affording the corresponding bis(indolyl)methanes in high yields (Table 2, entries 7, 14). It is noteworthy that the reaction of *N*-methylindole with aryl aldehydes gave the corresponding bis(indolyl)methanes in decreased yields (Table 2, entries 8–14). To expand the scope of the protocol, the reaction of various aryl aldehydes with 2-methylthiophene was also evaluated. The results are summarized in Table 3.

As shown in this table, good yields were obtained in GDE at 80 °C, except in the case of *p*-methyl-benzaldehyde (Table 3, entry 3). Surprisingly, applying these optimised conditions to perform the reaction of aryl aldehydes with 2-methylthiophene，resulted in a zero yield of the corresponding bis(thienyl)methanes, and in this case the reaction temperature must be changed, and 80 °C was the best choice. Steric effects also had an adverse influence on the reaction. For instance, 2-bromo-benzaldehyde gave a lower yield of 61% (Table 3, entry 3).

**Table 3:** RuCl_3_·3H_2_O-catalyzed reaction of 2-methyl thiophene with aryl aldehydes *^a^*.

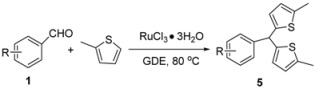				
Entry	Compounds	R	Time/h	Yield [%] *^b^*
1	**5a**	H	6.5	96
2	**5b**	*m*-CH_3_	6.0	90
3	**5c**	*p*-CH_3_	13	61
4	**5d**	*m*-OCH_3_	7.5	81
5	**5e**	*m*-Cl	13	84
6	**5f**	*o*-Br	5.5	75
7	**5g**	*m*-NO_2_	9.0	98

*^a^* The reaction was performed with aldehyde (0.5 mmol), 2-methyl thiophene (1.5 mmol) and RuCl_3_·3H_2_O (0.05 mmol) in 1 mL of GDE at 80 °C. *^b^* Isolated yield.

Nair has reported that 2-methylthiophene on reaction with benzaldehyde gave 70% of the corresponding bis(thienyl)methane using AuCl_3_/AgOTf as catalyst [27]. Compared to Nair’s method, the advantages of our procedure include the simplicity of the reaction procedure, as well as higher yields. In addition, the reaction of various aryl aldehydes with 2-methylfuran was also investigated. The results are summarized in Table 4.

**Table 4:** RuCl_3_·3H_2_O-catalyzed reaction of 2-methyl furan with aryl aldehydes *^a^*.

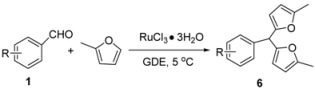				
Entry	Compounds	R	Time/days	Yield [%] *^b^*
1	**6a**	*m*-CH_3_	14	49
2	**6b**	*p*-CH_3_	14	52
3	**6c**	*m*-OCH_3_	14	50
4	**6d**	*m*-Cl	9	58
5	**6e**	*o*-Br	13	56
6	**6f**	*m*-NO_2_	6	79

*^a^* The reaction was performed with aldehyde (0.5 mmol), 2-methyl furan (6 mmol) and RuCl_3_·3H_2_O (0.05 mmol) in 1 mL of GDE at 5 °C^o^C. *^b^* Isolated yield.

Similarly, applying the previously optimized conditions to perform the reaction of *m*-methylbenzaldehyde with 2-methylfuran, resulted in a very low yield of the corresponding bis(fur-2-yl)methane. Fortunately, a mixture of *m*-methylbenzaldehyde and 2-methylfuran could be very slowly converted to the desired product in 49% yield after 14 days at 5 °C. Other aryl benzaldehydes also reacted well giving moderate yields under the same conditions (Table 4). Electron-withdrawing substituents on the aryl aldehyde were more beneficial for this transformation. For instance, *m*-nitro- benzaldehyde gave a higher reaction yield of 79% (Table 4, entry 6). To the best of our knowledge, the reports of such reactions of furans with aryl benzaldehydes are limited [27].

**Figure 1:**
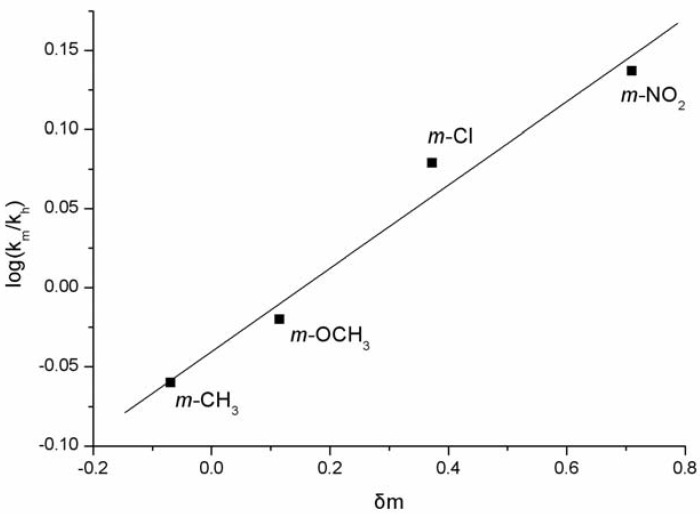
Hammett plot for C-3 substituted benzaldehydes.

A Hammett analysis was performed to probe the nature of this intriguing reaction of aryl aldehydes with *N*-methylindole. As can be observed from the plot for C-3 substituted benzaldehydes (Figure 1), a linear correlation between the ratio of reaction rates (k_n_ = rate constant of the reaction of benzaldehyde with *N*-methyl indole; k_m_ = rate constant of the reaction of aryl benzaldehyde with *N*-methyl indole; For the determination of r, the following expression was used: k_m_/k_n_ = log[1−xp/xr]/log[1−yp/yr], r = reaction constant; x_p_ = mmol product formed from substituted benzaldehyde; x_r _= mmol starting *N*-methyl indole placed in the reaction; y_p_ = mmol product formed from unsubstituted benzaldehyde; y_r_ = mmol *N*-methyl indole starting placed in the reaction.) and the substituent parameter (δ_m_) [28] was obtained, which provided a small, positive reaction constant (*ρ* = 0.26). This relatively small *ρ* value correlates to a slight dependence of the reaction on the polarizing influence of the aromatic substituents, which is indicative of a nucleophilic addition mechanism.

According to the literature [18,21,22,24], the following mechanism was proposed to account for the reaction of benzaldehyde with indole. The aldehyde was first activated by catalyst, then underwent an electrophilic substitution reaction at C-3 of an indole molecules to give mono(indolyl)methane **7**. After loss of water, intermediate **8** was generated. Compound **8** served as an electrophile to attack a second molecule of indole to form **2a**. To explore the RuCl_3_·3H_2_O-catalyzed reaction process, the reaction of mono(indolyl)methanes **7** with indole was performed in the presence of RuCl_3_·3H_2_O at r.t. Unfortunately, it was found that the reaction did not work, suggesting that **7** is not the intermediate of the RuCl_3_·3H_2_O-catalyzed reaction. The detailed mechanism has therefore not been clarified.

**Scheme 1:**
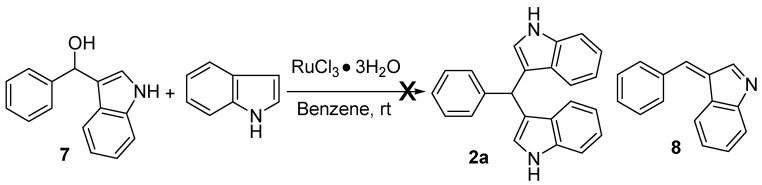
Thereactionof mono(indolyl)methanes with indole.

## 3. Experimental

### 3.1. General

Infrared spectra were measured with a Nicolet Avatar 360 FT-IR spectrometer using film KBr pellet techniques. ^1^H- and ^13^C-NMR spectra were recorded on a Bruker AV400 spectrometer at 400 and 100 MHz, respectively. Chemical shifts were reported in ppm relative to TMS. CDCl_3_ or DMSO-*d6* were used as the NMR solvents. GC-MS were recorded using a Finnigan Trace 2000 GC/MS system. Elemental analysis were carried out on a Perkin-Elmer 240B instrument. HRMS spectra were recorded on a Shimadzu LCMS-IT-TOF apparatus. Silica gel (300–400 mesh) was used for flash column chromatography, eluting (unless otherwise stated) with an ethyl acetate/petroleum ether (PE, b.p. 60–90 °C) mixture.

### 3.2. General Procedure for the Preparation of Bis(indolyl)Methanes ***2-4***

To a solution of aryl benzaldehyde (0.5 mmol) and RuCl_3_·3H_2_O (0.05 mmol) in benzene (1 mL) was added indole (1.0 mmol) under air atmosphere and the mixture was stirred at room temperature (monitored by TLC). Then, the reaction mixture was concentrated under reduced pressure. The residue was purified by flash chromatography on silica gel (eluent:EtOAc/PE = 1:4) to yield the corresponding product.

*3,3’-Bis-indolyl phenylmethane* (**2a**). Pink solid, mp: 126–127 °C (EtOAc/PE = 1:4) (lit [29], 125–127 °C). IR (KBr) ν_max_: 3417, 3065, 1513, 1454 cm^−1^. ^1^H-NMR (CDCl_3_): *δ* 7.82 (s, 2H, N–H), 7.43 (d, *J* = 7.9 Hz, 2H, Ar–H), 7.40–7.36 (m, 3H, Ar–H), 7.35–7.29 (m, 3H, Ar–H), 7.28–7.24 (m, 1H, Ar–H), 7.21 (dt, *J* = 0.8, 7.9 Hz, 2H, Ar–H), 7.05 (dt, *J* = 0.8, 7.9 Hz, 2H, Ar–H), 6.61 (d, *J* = 1.5 Hz, 2H, Ar–H), 5.92 (s, 1H). ^13^C-NMR (CDCl_3_): *δ* 144.0, 136.7, 128.8, 128.3, 127.1, 126.2, 123.7, 121.9, 120.0, 119.7, 119.2, 111.1, 40.2. MS (EI, 70 eV): *m/z* = 322 (M^+^, 20), 245 (75), 206 (100), 77 (10).

*3,3’-Bis-indolyl-(3-methylphenyl)methane* (**2b**). Pink solid, mp: 98–99 °C (EtOAc/PE = 1:4). IR (KBr) ν_max_: 3406, 3049, 1610, 1458, 1419, 745 cm^−1^. ^1^H-NMR (CDCl_3_): *δ* 7.88 (s, 2H, N–H), 7.42 (d, *J* = 7.9 Hz, 2H, Ar–H), 7.36 (d, *J* = 8.2 Hz, 2H, Ar–H), 7.20–7.14 (m, 5H, Ar–H), 7.05–6.99 (m, 3H, Ar–H), 6.66 (d, *J* = 1.6 Hz, 2H, Ar–H), 5.85 (s, 1H), 2.30 (s, 3H, CH_3_). ^13^C-NMR (CDCl_3_): *δ* 143.9, 137.6, 136.7, 129.5, 128.1, 127.1, 126.9, 125.8, 123.6, 121.9, 120.0, 119.8, 119.2, 111.0, 40.1, 21.5. MS (EI, 70 eV): *m/z* = 336 (M^+^, 30), 245 (100), 221 (30). Anal. calcd. for C_24_H_20_N_2_: C, 85.68; H, 5.99; N, 8.33. Found C, 85.30; H, 5.87; N, 8.05. 

*3,3’-Bis-indolyl-(4-methylphenyl)methane* (**2c**). Pink solid, mp: 93–95 °C (EtOAc/PE = 1:4) (lit [30], 94–96 °C). IR (KBr) ν_max_: 3410, 3046, 1457, 743 cm^−1^. ^1^H-NMR (CDCl_3_): *δ* 7.89 (s, 2H, N–H), 7.42 (d, *J* = 7.9 Hz, 2H, Ar–H), 7.36 (d, *J* = 8.2 Hz, 2H, Ar–H), 7.25 (d, *J* = 8.0 Hz, 2H, Ar–H), 7.18 (dt, *J* = 1.0, 8.2 Hz, 2H, Ar–H), 7.10 (d, *J* = 7.9 Hz, 2H, Ar–H), 7.02 (dt, *J* = 1.0, 8.0 Hz, 2H, Ar–H), 6.66 (dd, *J* = 2.2, 0.7 Hz, 2H, Ar-H), 5.87 (s, 1H), 2.34 (s, 3H, CH_3_). ^13^C-NMR (CDCl_3_): *δ* 141.0, 136.7, 135.5, 128.9, 128.6, 127.1, 123.5, 121.9, 120.0, 119.9, 119.2, 111.0, 39.8, 21.1. MS (EI, 70 eV): *m/z* = 336 (M^+^, 35), 245 (100), 220 (35), 116 (10). 

*3,3’-Bis-indolyl-(3-methoxyphenyl)methane* (**2d**). Pink solid, mp: 188.5–189.5 °C (EtOAc/PE = 1:4). IR (KBr) ν_max_: 3410, 3046, 2923, 1487, 1441, 1263, 1152, 1049, 745 cm^−1^. ^1^H-NMR (CDCl_3_): *δ* 7.91 (s, 2H, N–H), 7.42 (d, *J* = 7.8 Hz, 2H, Ar–H), 7.36 (d, *J* = 8.2 Hz, 2H, Ar–H), 7.22 (d, *J* = 7.8 Hz, 1H, Ar–H), 7.18 (dt, *J* = 0.9, 8.2 Hz, 2H, Ar–H), 7.02 (dt, *J* = 0.9, 7.8 Hz, 2H, Ar–H), 6.97 (d, *J* = 7.7 Hz, 1H, Ar–H), 6.93 (t, *J* = 2.2 Hz, 1H, Ar–H), 6.77 (dq, *J* = 0.6, 7.7 Hz, 1H, Ar–H), 6.68 (dd, *J* = 2.2, 0.6 Hz, 2H, Ar–H), 5.87 (s, 1H), 2.34 (s, 3H, CH_3_), 3.75 (s, 3H, OCH_3_). ^13^C-NMR (CDCl_3_): *δ* 159.6, 145.7, 136.7, 129.1, 127.1, 123.6, 121.9, 121.3, 119.9, 119.6, 119.2, 114.7, 111.3, 111.0, 55.1, 40.2. MS (EI, 70 eV): *m/z* = 352 (M^+^, 35), 337 (35), 321 (8), 245 (100), 130 (40). Anal. calcd for C_24_H_20_N_2_O: C, 81.79; H, 5.72; N, 7.95. Found C, 81.52; H, 5.33; N, 7.66. 

*3,3’-Bis-indolyl-(3-chlorophenyl)methane* (**2e**). Pink solid, mp: 64–68 °C (EtOAc/PE = 1:4). IR (KBr) ν_max_: 3412, 3046, 1458, 1418, 1094, 744 cm^−1^. ^1^H-NMR (CDCl_3_): *δ* 7.88 (s, 2H, N–H), 7.42–7.34 (m, 5H, Ar–H), 7.26–7.17 (m, 5H, Ar–H), 7.04 (dt, *J* = 0.8, 7.8 Hz, 2H, Ar–H), 6.62 (s, 2H, Ar–H), 5.86 (s, 1H). ^13^C-NMR (CDCl_3_): *δ* 146.2, 136.7, 134.0, 129.5, 128.8, 126.9, 126.8, 126.4, 123.6, 122.1, 119.7, 119.4, 119.0, 111.1, 40.0. MS (EI, 70 eV): *m/z* = 283 (25), 281 (100), 245 (80). Anal. calcd. for C_23_H_17_N_2_Cl: C, 77.41; H, 4.80; N, 7.85. Found C, 77.51; H, 4.67; N, 7.48.

*3,3’-Bis-indolyl-(2-bromophenyl)methane* (**2f**). Pink solid, mp: 89–91 °C (EtOAc/PE = 1:4). IR (KBr) ν_max_: 3411, 3043, 1443, 1022, 744 cm^−1^. ^1^H-NMR (CDCl_3_): *δ* 7.90 (s, 2H, N–H), 7.64 (d, *J* = 7.9 Hz, 1H, Ar–H), 7.42 (d, *J* = 7.9 Hz, 2H, Ar–H), 7.37 (d, *J* = 8.2 Hz, 2H, Ar–H), 7.25–7.14 (m, 4H, Ar–H), 7.10 (dt, *J* = 1.9, 7.9 Hz, 1H, Ar–H), 7.04 (dt, *J* = 0.9, 8.0 Hz, 2H, Ar–H), 6.62 (dd, *J* = 2.3, 0.9 Hz, 2H, Ar–H), 6.33 (s, 1H). ^13^C-NMR (CDCl_3_): *δ* 143.0, 136.7, 132.9, 130.5, 127.8, 127.3, 127.0, 124.8, 123.8, 122.0, 119.9, 119.3, 118.5, 111.1, 39.6. MS (EI, 70 eV): *m/z* = 351 (100), 349 (100), 245 (80). Anal. calcd. for C_23_H_17_N_2_Br: C, 68.84; H, 4.27; N, 6.98. Found C, 68.64; H, 4.05; N, 6.81. 

*3,3’-Bis-indolyl-(3-nitrophenyl)methane* (**2g**). Pink solid, mp: 262–264 °C (EtOAc/PE = 1:4) (lit [29], 265–266 °C). IR (KBr) ν_max_: 3410, 3053, 2924, 1524, 1455, 1346, 1092, 741 cm^−1^. ^1^H-NMR (CDCl_3_): *δ* 8.23 (t, *J* = 2.0 Hz, 1H, Ar–H), 8.10 (dq, *J* = 1.0, 8.2 Hz, 1H, Ar–H), 8.01 (s, 2H, N–H), 7.71 (d, *J* = 7.9 Hz, 1H, Ar–H), 7.46 (t, *J* = 7.9 Hz, 1H, Ar–H), 7.39 (d, *J* = 8.2 Hz, 2H, Ar–H), 7.37 (d, *J* = 7.9 Hz, 2H, Ar–H), 7.22 (dt, *J* = 0.9, 7.2 Hz, 2H, Ar–H), 7.04 (dt, *J* = 0.9, 7.2 Hz, 2H, Ar–H), 6.68 (dd, *J* = 2.0, 0.9 Hz, 2H, Ar–H), 6.01 (s, 1H). ^13^C-NMR (CDCl_3_): *δ* 148.5, 146.4, 136.7, 134.9, 129.2, 126.6, 123.7, 123.6, 122.3, 121.5, 119.6, 119.5, 118.3, 111.3, 40.0. MS (EI, 70 eV): *m/z* = 367 (100), 321 (10), 245 (85), 122 (20). Anal calcd. for C_23_H_17_N_3_O_2_: C, 75.19; H, 4.66; N, 11.44. Found C, 75.07; H, 4.36; N, 11.14. 

*3,3’-Bis-(N-methylindolyl)phenylmethane* (**3a**). Pink solid, mp: 185–187 °C (EtOAc/PE = 1:4). IR (KBr) ν_max_: 3046, 2930, 1607, 1474, 1329, 1125, 743 cm^−1^. ^1^H-NMR (CDCl_3_): *δ* 7.43 (d, *J* = 7.9 Hz, 2H, Ar–H), 7.39 (d, *J* = 8.6 Hz, 2H, Ar–H), 7.35–7.29 (m, 4H, Ar–H), 7.26-7.21 (m, 3H, Ar–H), 7.03 (dt, *J* = 0.8, 7.9 Hz, 2H, Ar–H), 6.57 (s, 2H, Ar–H), 5.93 (s, 1H), 3.71 (s, 6H, 2 × CH_3_). ^13^C-NMR (CDCl_3_): *δ* 144.5, 137.4, 128.7, 128.3, 128.2, 127.5, 126.0, 121.4, 120.1, 118.7, 118.3, 109.1, 40.1, 32.7. MS (EI, 70 eV): *m/z* = 350 (M^+^, 100), 273 (85), 220 (25), 130 (15). Anal. calcd. for C_25_H_22_N_2_: C, 85.68; H, 6.33; N, 7.99. Found C, 85.90; H, 6.58; N, 7.64. 

*3,3’-Bis-(N-methylindolyl)-(3-methylphenyl)methane* (**3b**). Pink waxy solid. IR (KBr) ν_max_: 3049, 2932, 1475, 1123, 737 cm^−1^. ^1^H-NMR (CDCl_3_): *δ* 7.42 (d, *J* = 7.9 Hz, 2H, Ar–H), 7.31 (d, *J* = 8.2 Hz, 2H, Ar–H), 7.25–7.14 (m, 5H, Ar–H), 7.07–6.99 (m, 3H, Ar–H), 6.56 (s, 2H, Ar–H), 5.87 (s, 1H), 3.70 (s, 6H, 2 × CH_3_), 2.32 (s, 3H, CH_3_). ^13^C-NMR (CDCl_3_): *δ* 144.4, 137.6, 137.4, 129.4, 128.2, 128.0, 127.5, 126.8, 125.7, 121.4, 120.1, 118.6, 118.4, 109.0, 40.0, 32.7, 21.6. MS (EI, 70 eV): *m/z* = 364 (M^+^, 95), 349 (85), 273 (100), 257 (25), 130 (20). Anal. calcd. for C_26_H_24_N_2_: C, 85.68; H, 6.64; N, 7.69. Found C, 85.30; H, 6.60; N, 7.36. 

*3,3’-Bis-(N-methylindolyl)-(4-methylphenyl)methane* (**3c**). Pink solid, mp: 146–148 °C (EtOAc/PE = 1:4). IR (KBr) ν_max_: 3050, 2928, 1470, 1125, 745 cm^−1^. ^1^H-NMR (CDCl_3_): *δ* 7.40 (d, *J* = 7.9 Hz, 2H, Ar–H), 7.30 (d, *J* = 8.2 Hz, 2H, Ar–H), 7.26–7.17 (m, 4H, Ar–H), 7.09 (d, *J* = 7.9 Hz, 2H, Ar–H), 7.00 (dt, *J* = 1.0, 7.9 Hz, 2H, Ar–H), 6.54 (s, 2H, Ar–H), 5.85 (s, 1H), 3.69 (s, 6H, 2 × CH_3_), 2.32 (s, 3H, CH_3_). ^13^C-NMR (CDCl_3_): *δ* 141.4, 137.4, 135.4, 128.9, 128.5, 128.2, 127.5, 121.4, 120.1, 118.6, 118.5, 109.0, 39.6, 32.6, 21.1. MS (EI, 70 eV): *m/z* = 364 (M^+^, 15), 273 (100), 257 (60), 130 (60). Anal. calcd. for C_26_H_24_N_2_: C, 85.68; H, 6.64; N, 7.69. Found C, 85.44; H, 6.96; N, 7.32.

*3,3’-Bis-(N-methylindolyl)-(3-methoxyphenyl)methane* (**3d**). Pink solid, mp: 149–151 °C (EtOAc/PE = 1:4). IR (KBr) ν_max_: 3054, 2930, 1478, 1256, 1135, 740 cm^−1^. ^1^H-NMR (CDCl_3_): *δ* 7.42 (d, *J* = 7.9 Hz, 2H, Ar–H), 7.30 (d, *J* = 8.2 Hz, 2H, Ar–H), 7.22 (t, *J* = 7.9 Hz, 3H, Ar–H), 7.02 (dt, *J* = 0.9, 7.9 Hz, 2H, Ar–H), 6.97 (d, *J* = 7.9 Hz, 1H, Ar–H), 6.94 (t, *J* = 2.0 Hz, 1H, Ar–H), 6.78 (dd, *J* = 8.2, 2.0 Hz, 1H, Ar–H), 6.57 (s, 2H, Ar–H), 5.87 (s, 1H), 3.76 (s, 3H, OCH_3_), 3.70 (s, 6H, 2 × CH_3_). ^13^C-NMR (CDCl_3_): *δ* 159.6, 146.2, 137.4, 129.1, 128.2, 127.5, 121.4, 121.3, 120.0, 118.6, 118.1, 114.8, 111.1, 109.0, 55.1, 40.1, 32.7. MS (EI, 70 eV): *m/z* = 380 (M^+^, 65), 365 (85), 349 (30), 273 (100), 130 (25). Anal. calcd. for C_26_H_24_N_2_O: C, 82.07; H, 6.36; N, 7.36. Found C, 81.72; H, 5.96; N, 6.99. 

*3,3’-Bis-(N-methylindolyl)-(3-chlorophenyl)methane* (**3e**). Pink solid, mp: 195–197 °C (EtOAc/PE = 1:4). IR (KBr) ν_max_: 3051, 2930, 1458, 1420, 1094, 743 cm^−1^. ^1^H-NMR (CDCl_3_): *δ* 7.45 (t, *J* = 7.9 Hz, 2H, Ar–H), 7.42 (s, 1H), 7.37 (d, *J* = 8.2 Hz, 2H, Ar–H), 7.36-7.25 (m, 5H, Ar–H), 7.10 (dt, *J* = 0.7, 7.9 Hz, 2H, Ar–H), 6.61 (s, 2H, Ar–H), 5.94 (s, 1H), 3.73 (s, 6H, 2 × CH_3_). ^13^C-NMR (CDCl_3_): *δ* 146.8, 137.5, 134.1, 129.5, 128.8, 128.3, 127.3, 127.0, 126.4, 121.6, 119.9, 118.9, 117.5, 109.2, 39.9, 32.7. MS (EI, 70 eV): *m/z* = 386 (M^+^, 20), 384 (M^+^, 60), 371 (5), 369 (15), 273 (100). Anal. calcd. for C_25_H_21_N_2_Cl: C, 78.01; H, 5.50; N, 7.28. Found C, 77.80; H, 5.50; N, 7.16. 

*3,3’-Bis-(N-methylindolyl)-(2-bromophenyl)methane* (**3f**). Pink solid, mp: 247–249 °C (EtOAc/PE = 1:4). IR (KBr) ν_max_: 3046, 2926, 1457, 1227, 1023, 792 cm^−1^. ^1^H-NMR (CDCl_3_): *δ* 7.63 (dd, *J* = 7.9, 1.2 Hz, 1H, Ar–H), 7.42 (d, *J* = 7.9 Hz, 2H, Ar–H), 7.31 (d, *J* = 8.2 Hz, 2H, Ar–H), 7.28–7.20 (m, 3H, Ar–H), 7.17 (dt, *J* = 1.2, 7.6 Hz, 1H, Ar–H), 7.09 (dt, *J* = 1.8, 7.6 Hz, 1H, Ar–H), 7.03 (dt, *J* = 0.9, 7.9 Hz, 2H, Ar–H), 6.51 (s, 2H, Ar–H), 6.33 (s, 1H), 3.70 (s, 6H, 2 × CH_3_). ^13^C-NMR (CDCl_3_): *δ* 143.4, 137.5, 132.8, 130.5, 128.5, 127.7, 127.4, 127.2, 124.8, 121.5, 120.0, 118.7, 117.0, 109.1, 39.4, 32.7. MS (EI, 70 eV): *m/z* = 430 (M^+^, 20), 428 (M^+^, 20), 350 (55), 273 (100), 130 (30). Anal. calcd. for C_25_H_21_N_2_Br: C, 69.94; H, 4.93; N, 6.52. Found C, 69.82; H, 4.55; N, 6.54. 

*3,3’-Bis-(N-methylindolyl)-(3-nitrophenyl)methane* (**3g**). Yellow solid, mp: 157–159 °C (EtOAc/PE = 1:4). IR (KBr) ν_max_: 3063, 2926, 1525, 1474, 1349, 743 cm^−1^. ^1^H-NMR (CDCl_3_): *δ* 8.27 (t, *J* = 1.9 Hz, 1H, Ar–H), 8.12 (dd, *J* = 8.2, 1.9 Hz, 1H, Ar–H), 7.74 (d, *J* = 7.9 Hz, 1H, Ar–H), 7.47 (t, *J* = 7.9 Hz, 1H, Ar–H), 7.41 (d, *J* = 7.9 Hz, 2H, Ar–H), 7.38 (d, *J* = 8.2 Hz, 2H, Ar–H), 7.28 (dt, *J* = 0.9, 7.9 Hz, 2H, Ar–H), 7.18 (dt, *J* = 0.9, 7.9 Hz, 2H, Ar–H), 6.61 (s, 2H, Ar–H), 6.05 (s, 1H), 3.75 (s, 6H, 2 × CH_3_). ^13^C-NMR (CDCl_3_): *δ* 148.5, 146.9, 137.5, 134.9, 129.1, 128.4, 127.1, 123.6, 121.9, 121.4, 119.7, 119.0, 116.8, 109.4, 40.0, 32.8. MS (EI, 70 eV): *m/z* = 395 (M^+^, 80), 380 (5), 349 (5), 273 (100), 122 (5). Anal. calcd. for C_25_H_21_N_3_O_2_: C, 75.93; H, 5.35; N, 10.63. Found C, 75.76; H, 4.98; N, 10.54.

*3,3’-Bis-(2-methylindolyl)-(3-methylphenyl)methane* (**4a**). Pink solid, mp: 181–184 °C (EtOAc/PE = 1:4). IR (KBr) ν_max_: 3383, 2915, 1677, 1607, 1459, 740 cm^−1^. ^1^H-NMR (DMSO-*d6*): *δ* 10.71 (s, 2H, N–H), 7.19 (d, *J* = 8.0 Hz, 2H, Ar–H), 7.11 (t, *J* = 8.0 Hz, 1H, Ar–H), 7.03–6.96 (m, 3H, Ar–H), 6.87 (dt, *J* = 0.9, 8.0 Hz, 2H, Ar–H), 6.81 (d, *J* = 8.0 Hz, 2H, Ar–H), 6.66 (dt, *J* = 0.9, 8.0 Hz, 2H, Ar–H), 5.87 (s, 1H), 2.19 (s, 3H, CH_3_), 2.04 (s, 6H, 2 × CH_3_). ^13^C-NMR (DMSO-*d6*): *δ* 144.65, 137.20, 135.48, 132.44, 129.81, 128.76, 128.20, 126.88, 126.24, 119.93, 118.92, 118.34, 112.69, 110.73, 39.00, 21.64, 12.38. MS (EI, 70 eV): *m/z* = 364 (M^+^, 15), 349 (100), 234 (40), 130 (70). Anal. calcd. for C_26_H_24_N_2_: C, 85.68; H, 6.64; N, 7.69. Found C, 85.50; H, 6.91; N, 7.45. 

*3,3’-Bis-(2-methylindolyl)-(3-methoxylphenyl)methane* (**4b**). Pink solid, mp: 147–150 °C (EtOAc/PE = 1:4). IR (KBr) ν_max_: 3385, 1594, 1459, 1147, 744 cm^−1^. ^1^H-NMR (DMSO-*d6*): *δ* 10.73 (s, 2H, N–H), 7.21 (d, *J* = 8.0 Hz, 2H, Ar–H), 7.16 (t, *J* = 8.0 Hz, 1H, Ar–H), 6.88 (dt, *J* = 0.9, 8.0 Hz, 2H, Ar–H), 6.83 (d, *J* = 8.0 Hz, 2H, Ar–H), 6.79–6.72 (m, 3H, Ar–H), 6.67 (dt, *J* = 0.9, 8.0 Hz, 2H, Ar–H), 5.88 (s, 1H), 3.62 (s, 3H, OCH_3_), 2.07 (s, 6H, 2 × CH_3_). ^13^C-NMR (DMSO-*d6*): *δ* 159.55, 146.39, 135.49, 132.47, 129.31, 128.71, 121.71, 119.97, 118.93, 118.36, 115.48, 112.57, 110.98, 110.75, 55.27, 39.05, 12.36. MS (EI, 70 eV): *m/z* = 380 (M^+^, 95), 365 (35), 349 (45), 273 (100), 130 (35). Anal. calcd. for C_26_H_24_N_2_O: C, 82.07; H, 6.36; N, 7.36. Found C, 81.85; H, 6.02; N, 7.17.

### 3.3. General Procedure for the Preparation of Bis(thienyl)methanes ***5a-5g***

To a solution of aryl benzaldehyde (0.5 mmol) and RuCl_3_·3H_2_O (0.05 mmol ) in ethylene glycol dimethyl ether (1 mL) was added 2-methylthiophene (1.0 mmol) under air atmosphere and the mixture was stirred at 80 °C (monitored by TLC). Then, the reaction mitxure was concentrated under reduced pressure. The residue was purified by flash chromatography on silica gel (eluent: EtOAc/PE = 1:8) to yield the corresponding product. 

*5,5’-Bis-(2-methylthienyl)phenylmethane* (**5a**). Yellow waxy solid. IR (KBr) ν_max_: 3059, 2919, 1525, 1448, 1225, 794 cm^−1^. ^1^H-NMR (MHz, CDCl_3_): *δ* 7.33–7.28 (m, 4H), 7.27–7.21 (m, 1H), 6.61–6.55 (m, 4H), 5.67 (s, 1H), 2.41 (s, 6H, 2 × CH_3_). ^13^C-NMR (CDCl_3_): *δ* 145.3, 143.8, 139.1, 128.4, 128.3, 127.0, 125.7, 124.5, 47.8, 15.4. MS (EI, 70 eV): *m/z* = 284 (M^+^, 100), 269 (95), 207 (50), 187 (20), 97 (5), 77 (5). HRESIMS calcd. for [C_17_H_16_S_2_ + H]^+^: 285.4469; found: 285.4466. 

*5,5’-Bis-(2-methylthienyl)-(3-methylphenyl)methane* (**5b**). Yellow waxy solid. IR (KBr) ν_max_: 3058, 2919, 2859, 1446, 800, 755 cm^−1^. ^1^H-NMR (CDCl_3_): *δ* 7.19 (t, *J* = 7.5 Hz, 1H), 7.13–7.03 (m, 3H), 6.58 (dd, *J* = 0.5, 3.5 Hz, 2H), 6.54 (dd, *J* = 1.0, 3.5 Hz, 2H), 5.66 (s, 1H), 2.44 (s, 6H, 2 × CH_3_), 2.35 (s, 3H, CH_3_). ^13^C-NMR (CDCl_3_): *δ* 145.4, 143.7, 139.0, 138.0, 129.0, 128.3, 127.8, 125.6, 125.3, 124.5, 47.8, 21.5, 15.4. MS (EI, 70 eV): *m/z* = 298 (M^+^, 98), 283 (100), 201 (15), 91 (5), 77 (5). HRESIMS calcd. for [C_18_H_18_S_2_ + H]^+^: 299.4735; found: 299.4733.

*5,5’-Bis-(2-methylthienyl)-(4-methylphenyl)methane* (**5c**). Yellow waxy solid. IR (KBr) ν_max_: 3062, 2920, 1533, 1448, 745 cm^−1^. ^1^H-NMR (CDCl_3_): *δ* 7.24 (d, *J* = 7.9 Hz, 2H), 7.16 (d, *J* = 7.9 Hz, 2H), 6.63 (d, *J* = 3.4 Hz, 2H), 6.61 (d, *J* = 3.4 Hz, 2H), 5.68 (s, 1H), 2.45 (s, 6H, 2 × CH_3_), 2.37 (s, 3H, CH_3_). ^13^C-NMR (CDCl_3_): *δ* 145.5, 140.9, 138.9, 136.5, 129.1, 128.1, 125.5, 124.4, 47.4, 21.0, 15.3. MS (EI, 70 eV): *m/z* = 298 (M^+^, 90), 283 (100), 201 (20), 91 (5), 77 (5). Anal. calcd. for C_18_H_18_S_2_: C, 72.43; H, 6.08. Found C, 72.80; H, 6.43. 

*5,5’-Bis-(2-methylthienyl)-(3-methoxyphenyl)methane* (**5d**). Yellow waxy solid. IR (KBr) ν_max_: 2922, 1599, 1487, 1448, 1265, 1156, 1046 cm^−1^. ^1^H-NMR (CDCl_3_): *δ* 7.26 (t, *J* = 7.9 Hz, 1H), 6.94 (d, *J* = 8.1 Hz, 1H), 6.89 (t, *J* = 2.0 Hz, 1H), 6.82 (dd, *J* = 0.6, 8.1 Hz, 1H), 6.63 (dd, *J* = 0.5, 3.5 Hz, 2H), 6.58 (dd, *J* = 1.0, 3.5 Hz, 2H), 5.67 (s, 1H), 3.80 (s, 3H, OCH_3_), 2.44 (s, 6H, 2 × CH_3_). ^13^C-NMR (CDCl_3_): *δ* 159.6, 145.4, 145.0, 139.1, 129.4, 125.7, 124.5, 120.8, 114.3, 112.1, 55.2, 47.8, 15.4. MS (EI, 70 eV): *m/z* = 314 (M^+^, 100), 299 (15), 283 (10), 207 (90), 122 (15). Anal. calcd. for C_18_H_18_S_2_O: C, 68.75; H, 5.77. Found C, 68.91; H, 5.84. 

*5,5’-Bis-(2-methylthienyl)-(3-chlorophenyl)methane* (**5e**). Yellow waxy solid. IR (KBr) ν_max_: 3063, 2919, 1473, 1262, 1095, 1034, 802 cm^−1^. ^1^H-NMR (CDCl_3_): *δ* 7.33 (s, 1H), 7.28–7.21 (m, 3H), 6.64–6.59 (m, 4H), 5.68 (s, 1H), 2.46 (s, 6H, 2 × CH_3_). ^13^C-NMR (CDCl_3_): *δ* 145.8, 144.3, 139.4, 134.3, 129.7, 128.5, 127.2, 126.6, 125.9, 124.7, 47.4, 15.4. MS (EI, 70 eV): *m/z* = 320 (M^+^, 24), 318 (M^+^, 99), 305 (25), 303 (100), 283 (10), 223 (8), 221 (25), 207 (99), 113 (7), 111 (20). Anal. calcd. for C_17_H_15_S_2_Cl: C, 64.03; H, 4.74. Found C, 64.06; H, 4.92. 

*5,5’-Bis-(2-methylthienyl)-(2-bromophenyl)methane* (**5f**). Yellow waxy solid. IR (KBr) ν_max_: 3063, 2923, 2856, 1442, 1229, 1028, 795 cm^−1^. ^1^H-NMR (CDCl_3_): *δ* 7.55 (dd, *J* = 7.9, 1.2 Hz, 1H), 7.32 (dd, *J* = 7.9, 1.8 Hz, 1H), 7.25 (dt, *J* = 1.2, 7.9 Hz, 1H), 7.10 (dt, *J* = 1.8, 7.9 Hz, 1H), 6.59–6.54 (m, 4H), 6.13 (s, 1H), 2.42 (s, 6H, 2 × CH_3_). ^13^C-NMR (CDCl_3_): *δ* 143.8, 143.0, 139.3, 132.9, 130.1, 128.6, 127.6, 126.2, 124.6, 124.4, 46.7, 15.4. . MS (EI, 70 eV): *m/z* = 364 (M^+^, 100), 362 (M^+^, 100), 349 (60), 347 (60), 283 (25), 207 (60), 97 (25). Anal. calcd. for C_17_H_15_S_2_Br: C, 56.20; H, 4.16. Found C, 56.53; H, 4.47. 

*5,5’-Bis-(2-methylthienyl)-(3-nitrophenyl)methane* (**5g**). Yellow waxy solid. IR (KBr) ν_max_: 3061, 2918, 1529, 1350, 804 cm^−1^. ^1^H-NMR (CDCl_3_): *δ* 8.18 (t, *J* = 1.9 Hz, 1H), 8.13 (dq, *J* = 0.9, 8.2 Hz, 1H), 7.66 (d, *J* = 7.9 Hz, 1H), 7.50 (t, *J* = 7.9 Hz, 1H), 6.63-6.58 (m, 4H), 5.80 (s, 1H), 2.44 (s, 6H, 2 × CH_3_). ^13^C-NMR (CDCl_3_): *δ* 148.4, 145.9, 143.4, 139.9, 134.4, 129.4, 126.2, 124.8, 123.3, 122.1, 47.3, 15.4. MS (EI, 70 eV): *m/z* = 329 (M^+^, 100), 314 (96), 283 (5), 232 (5), 207 (70), 97 (5), 77 (5). Anal. calcd. for C_17_H_15_NO_2_S_2_: C, 61.98; H, 4.59; N, 4.25. Found C, 62.37; H, 4.63; N, 4.45.

### 3.4. General Procedure for the Preparation of Bis(fur-2-yl)methanes ***6a-6f***

To a cooled (0 °C) solution of aryl benzaldehyde (0.5 mmol) and RuCl_3_·3H_2_O (0.05 mmol) in ethylene glycol dimethyl ether (1 mL) was added 2-methylfuran (6.0 mmol) under air atmosphere and the mixture was placed into refrigerator to stay without stirring at 5 °C. The mixture was shaken for several seconds every day to ensure homodispersity (monitored by TLC). The reaction mitxure was then concentrated under reduced pressure. The residue was purified by flash chromatography on silica gel (eluent: EtOAc/PE = 1:8) to yield the corresponding product.

*5,5’-Bis-(2-methylfuryl)-(3-methylphenyl)methane* (**6a**). Waxy solid. IR (KBr) ν_max_: 2922, 1608, 1449, 1137, 1021, 779 cm^−1^. ^1^H-NMR (CDCl_3_): *δ* 7.24 (t, *J* = 7.5 Hz, 1H), 7.11 (s, 2H), 7.09 (s, 1H), 5.93 (d, *J* = 3.3 Hz, 2H), 5.91 (d, *J* = 3.3 Hz, 2H), 5.35 (s, 1H), 2.37 (s, 3H, CH_3_), 2.29 (s, 6H, 2 × CH_3_). ^13^C-NMR (CDCl_3_): *δ* 153.0, 151.4, 139.9, 138.0, 129.1, 128.3, 127.8, 125.5, 108.1, 106.1, 45.1, 21.5, 13.6. MS (EI, 70 eV): *m/z* = 266 (M^+^, 60), 251 (100), 175 (60). HRESIMS calcd. for [C_18_H_18_O_2_ + H]^+^: 267.3423; found: 267.3417. 

*5,5’-Bis-(2-methylfuryl)-(4-methylphenyl)methane* (**6b**). Waxy solid. IR (KBr) ν_max_: 2922, 1607, 1510, 1448, 1130, 1014, 775 cm^−1^. ^1^H-NMR (CDCl_3_): *δ* 7.20 (d, *J* = 8.2 Hz, 2H), 7.16 (d, *J* = 8.2 Hz, 2H), 5.92 (d, *J* = 3.5 Hz, 2H), 5.90 (d, *J* = 3.5 Hz, 2H), 5.35 (s, 1H), 2.37 (s, 3H, CH_3_), 2.28 (s, 6H, 2 × CH_3_). ^13^C-NMR (CDCl_3_): *δ* 153.1, 151.4, 137.1, 136.5, 129.2, 128.3, 108.1, 106.1, 44.8, 21.1, 13.6. MS (EI, 70 eV): *m/z* = 266 (M^+^, 100), 251 (20), 185 (15), 175 (55). HRESIMS calcd. for [C_18_H_18_O_2_ + H]^+^: 267.3423; found: 267.3422.

*5,5’-Bis-(2-methylfuryl)-(3-methoxyphenyl)methane* (**6c**). Waxy solid. IR (KBr) ν_max_: 2922, 1600, 1262, 1151, 771 cm^−1^. ^1^H-NMR (CDCl_3_): *δ* 7.26 (t, *J* = 7.8 Hz, 1H), 6.87 (d, *J* = 7.8 Hz, 1H), 6.84–6.80 (m, 2H), 5.91 (d, *J* = 3.8 Hz, 2H), 5.89 (d, *J* = 3.8 Hz, 2H), 5.33 (s, 1H), 3.79 (s, 3H, OCH_3_), 2.27 (s, 6H, 2 × CH_3_). ^13^C-NMR (CDCl_3_): *δ* 159.7, 152.7, 151.4, 141.6, 129.3, 120.8, 114.3, 112.2, 108.2, 106.1, 55.1, 45.1, 13.6. MS (EI, 70 eV): *m/z* = 282 (M^+^, 100), 251 (80), 175 (60). HRESIMS calcd. for [C_18_H_19_O_3_ + H]^+^: 283.3417; found: 283.3411.

*5,5’-Bis-(2-methylfuryl)-(3-chlorophenyl)methane* (**6d**). Waxy solid. IR (KBr) ν_max_: 2923, 1624, 1437, 1131 cm^−1^. ^1^H-NMR (CDCl_3_): *δ* 7.26–7.23 (m, 3H), 7.16–7.14 (m, 1H), 5.91 (d, *J* = 3.2 Hz, 2H), 5.89 (d, *J* = 3.2 Hz, 2H), 5.32 (s, 1H), 2.26 (s, 6H, 2 × CH_3_). ^13^C-NMR (CDCl_3_): *δ* 152.0, 151.7, 142.0, 134.2, 129.7, 128.5, 127.2, 126.6, 108.5, 106.2, 44.7, 13.6. MS (EI, 70 eV): *m/z* = 288 (M^+^, 20), 286 (M^+^, 60), 273 (M^+^, 5), 271 (M^+^, 15), 175 (100). HRESIMS calcd. for [C_17_H_16_O_2_Cl + H]^+^: 287.7607; found: 287.7601. 

*5,5’-Bis-(2-methylfuryl)-(2-bromophenyl)methane* (**6e**). Waxy solid. IR (KBr) ν_max_: 2926, 1462, 1131, 1021, 747 cm^−1^. ^1^H-NMR (CDCl_3_): *δ* 7.58 (dd, *J* = 7.9, 1.0 Hz, 1H), 7.27 (dt, *J* = 1.0, 7.9 Hz, 1H), 7.21 (dd, *J* = 7.9, 1.8 Hz, 1H), 7.14 (dt, *J* = 1.8, 7.9 Hz, 1H), 5.91 (d, *J* = 3.0 Hz, 2H), 5.86 (d, *J* = 3.0 Hz, 2H), 5.83 (s, 1H), 2.27 (s, 6H, 2 × CH_3_). ^13^C-NMR (CDCl_3_): *δ* 151.7, 151.6, 139.1, 132.9, 130.1, 128.5, 127.5, 124.5, 108.8, 106.1, 44.4, 13.6. MS (EI, 70 eV): *m/z* = 332 (M^+^, 100), 330 (M^+^, 100), 317 (20), 315 (20), 175 (65). HRESIMS calcd. for [C_17_H_16_O_2_Br + H]^+^: 332.2117; found: 332.2109. 

*5,5’-Bis-(2-methylfuryl)-(3-nitrophenyl)methane* (**6f**). Waxy solid. IR (KBr) ν_max_: 2922, 1528, 1348, 1132, 781 cm^−1^. ^1^H-NMR (CDCl_3_): *δ* 8.13–8.11 (m, 2H), 7.59 (d, *J* = 7.9 Hz, 1H), 7.48 (dt, *J* = 2.3, 7.9 Hz, 1H), 5.94 (d, *J* = 3.0 Hz, 2H), 5.92 (d, *J* = 3.0 Hz, 2H), 5.44 (s, 1H), 2.25 (s, 6H, 2 × CH_3_). ^13^C-NMR (CDCl_3_): *δ* 152.1, 151.1, 148.4, 142.2, 134.6, 129.3, 123.4, 122.2, 108.8, 106.3, 44.7, 13.6. MS (EI, 70 eV): *m/z* = 297 (M^+^, 90), 282 (15), 175 (100). HRESIMS calcd. for [C_17_H_16_NO_4_ + H]^+^: 298.3132; found: 298.3129. 

## 4. Conclusions

In summary, RuCl_3_·3H_2_O has been demonstrated to be a mild and effective catalyst for the reactions of aryl aldehydes with indoles, 2-methylthiophenes, and 2-methylfurans, respectively. The catalyzed reactions produced the corresponding bis(indolyl)methanes, bis(thienyl)methanes, and bis(fur-2-yl)methanes in moderate to excellent yields. The procedure offers several advantages, including mild reaction conditions and simple experimental and isolation procedures, which makes it is a useful and attractive process for the synthesis of bis(indolyl)methanes, bis(thienyl)methanes and bis(fur-2-yl)methanes. 
